# A CRITICAL REVIEW OF DISCHARGE PLANNING AND DECISION-MAKING EXPERIENCE OF DEMENTIA FAMILY CAREGIVERS

**DOI:** 10.1093/geroni/igae098.0667

**Published:** 2024-12-31

**Authors:** Kwaku Duah Oppong, Jung Kwak

**Affiliations:** The University of Texas Austin, Austin, Texas, United States; The University of Texas Austin, Austin, Texas, United States

## Abstract

Persons living with dementia (PLWD) are significantly more likely to be hospitalized and experience poorer outcomes than their non-dementia peers. Despite available evidence-based care transition interventions, few are based on the experiences and perspectives of PLWDs and their family caregivers. To inform development of person-and family-centered care transition support for PLWDs and family caregivers, a systematic review focused on their decision-making roles and support received from hospital teams was conducted by retrieving studies published between 2000 and 2024 from PubMed, CINAHL, and Embase. Four papers were included for full review, and the rigor of each paper was assessed using the Mixed-Methods Assessment Tool. All four studies were qualitative, cross-sectional, and atheoretical, with only one study conducted in the United States. The sample size ranged from 15 to 30 and consisted of family caregivers and PLWDs recruited via chart reviews and hospital units. No study reported on the types of decisions that family caregivers were asked to make. Overall themes on information and support received were: lack of care coordination and inclusion in discharge planning, the discharge planning process often rushed, caregivers feeling unsupported by healthcare providers, and health providers’ inability to meet family caregivers’ informational needs and preferences. Surprisingly, few empirical studies are available, with no studies examining shared decision-making between PLWDs, caregivers, and providers. Existing studies suggest lack of information and support by the healthcare team as a major challenge for these families. Further studies that are theory-driven, with methodological rigor and racial/ethnically diverse PLWDs and caregivers are warranted.

